# A pilot single centre, double blind, placebo controlled, randomized, parallel study of Calmagen® dermaceutical cream and lotion for the topical treatment of tinea and onychomycosis

**DOI:** 10.1186/s12906-017-1970-2

**Published:** 2017-09-18

**Authors:** Manoj Parekh, Girisha Ramaiah, Prachi Pashilkar, Ranjani Ramanujam, Peter Johnston, Leodevico L. Ilag

**Affiliations:** 1 The Apollo Clinic, Frazer Town, Bangalore, India; 2 Mahaveer Jain Hospital, Bangalore, India; 3 Manipal AcuNova Ltd., Bangalore, India; 4 SPRIM India, Bangalore, India; 5 Biovite Australia Pty Ltd., Burleigh Heads, 4219 Queensland Australia; 6 Xerion Limited, Brighton, Victoria 3186 Australia; 7 Bio21 Molecular Science and Biotechnology Institute, University of Melbourne, Melbourne, 3010 Victoria Australia

**Keywords:** Superficial dermatophytosis, Tinea, Onychomycosis, Calmagen®, AMYCOT®, Natural antifungal, Cyanobacterium-derived anti-fungal

## Abstract

**Background:**

Most of the current anti-fungal treatments are chemical-based, fungistatic, have low efficacy in the treatment of tinea and toxicity concerns, while onychomycosis remains recalcitrant to most antifungal therapies. The study aimed to establish the fungicidal, efficacy and safety profile of Calmagen® dermaceutical cream and lotion containing AMYCOT® as a topical treatment in patients with severe to very severe presentations of fungal skin (tinea) and nail infections (onychomycosis).

**Methods:**

A randomized, placebo-controlled, double blind, parallel, single centre study was conducted on 28 subjects with severe to very severe tinea or onychomycosis. All patients were randomized in a ratio of 1:1 for treatment or placebo group. Subjects in the treatment arm received Calmagen® cream or lotion, while subjects in the placebo arm received a similar inert topical preparation. Tinea subjects were treated with cream for four weeks, while onychomycosis subjects were treated with lotion for 12 weeks. Mycological cure, the primary endpoint, was assessed by three parameters: KOH (potassium hydroxide) smear, fungal culture and live spore count. Clinical cure was defined as Investigator Global Assessment (IGA) response of ‘cleared’ or ‘excellent’.

**Results:**

All three parameters constituting mycological cure were confirmed in 92.8% (13/14) of subjects in the treatment arm, while all 14 subjects in the placebo arm remained positive for KOH smear. Calmagen® cream and lotion treatment showed a significant improvement in all three parameters: KOH smear, (95% CI (Calmagen): 79.4, 100.0; 95% CI (placebo): 0.0, 0.0; *p* < 0.0001); fungal culture (95% CI (Calmagen); 100.0, 100.0; 95% CI (Placebo): 17.0, 100.0; *p* < 0.0019); and live spore count (95% CI (Calmagen): 100.0, 100.0; 95% CI (Placebo): 17.0, 100.0; *p* < 0.0019). Clinical cure was achieved in all subjects in the treatment arm while none in the placebo arm were clinically cured. No treatment-related adverse effects were observed in either group.

**Conclusions:**

The Calmagen® cream and lotion containing AMYCOT® represent a potentially safe and efficacious natural alternative in the treatment of Tinea and onychomycosis.

**Trial registration:**

This trial has been registered with the clinical trial registry-India (CTRI; registration number: CTRI/2012/03/002522).

## Background

Dermatophytosis is the most common type of superficial fungal infection, adversely affecting the quality of life of individuals across all age groups, estimated to be 20–25% of the global population [1–4]. Currently available antifungal drugs have varied mechanisms of action affecting synthesis of membrane/cell-wall components (echinocandins), membrane permeability (amphoterecin-B, azoles, allylamines), synthesis of nucleic acids (flucytosine), and microtubule/mitotic spindle function (griseofulvin) [5, 6]. Agents, which disrupt the cell wall/membrane are generally fungicidal while inhibitors of fungal cell division are fungistatic. The fungicidal property of antifungals seems to depend on the minimum inhibitory concentrations (MIC) [7].

There are several oral or topical antifungal agents for the management of dermatophytosis [1]. However, dermatophytosis usually requires long-term therapy with allylamines (e.g. terbinafine) and azoles (e.g. ketoconazole, miconazole) [2]. Dermatophytosis is often successfully treated with topical antifungal agents. However, if not properly treated, these infections may become chronic, requiring oral antifungal drugs, which are often associated with hepatotoxicity [8]. Furthermore, complications like bacterial superinfection, lichenification and maceration can occur. Therefore, there is a need for prompt and effective treatment of dermatophytosis [9].

Of all dermatophytoses, onychomycosis remains resistant to most antifungal therapies. Currently available topical therapies are not very effective because of poor penetrability through the nail or being fungistatic. However, the US FDA has recently approved tavaborole and efinaconazole for topical use in treatment of mild to moderate onychomycosis [10, 11].

Calmagen®, a novel antifungal cream and lotion, has shown promising results in preclinical studies and medically supervised non-randomised human trials. It is listed with the Australian Therapeutics Goods Authority and approved by Health Canada under the brand, Mycolixin®.

The active component of Calmagen® is AMYCOT®, a bioactive extract, derived from *Arthospira maxima* (Spirulina), a filamentous cyanobacterium used as food for centuries. Extracts of *Arthospira maxima* have been documented to possess antimicrobial activities [12]. AMYCOT® has been extracted through a proprietary process of physiologically and mechanically stressing the cyanobacterium, which induces release of molecules with antimicrobial activities [13].

In vitro studies have shown that AMYCOT® is effective against fungi such as *Trichophyton mentagrophytes, Trichophyton rubrum, Epidermophyton floccosum*, including the yeasts, *Candida albicans* and *Malassezia furfur* and the bacterium, *Propionibacterium acnes* [13]. Preliminary chemical analysis of AMYCOT® (unpublished results) shows a diversity of molecules present in the extract – fatty acids, carbohydrate and proteins – all of which are known to have anti-fungal properties [12]. One of AMYCOT®‘s unique fungicidal properties includes enzymatic hydrolysis of chitin, the structural component of the fungal cell wall [13]. The actual molecule(s) responsible for chitin hydrolysis has not yet been isolated. However, AMYCOT®’s potent fungicidal property can possibly be attributed to the synergistic effects of the aforementioned bioactive molecules.

Efficacy was further demonstrated against acne, deep nail bed infection, chronic varicose ulcers, and burns. Additional studies confirmed that AMYCOT® was effective against tinea (unpublished results) and onychomycosis [14].

The current study was conducted to establish the fungicidal, efficacy and safety profiles of Calmagen® dermaceutical cream and lotion as a topical treatment in patients with severe to very severe presentations of tinea and onychomycosis.

## Methods

This clinical study was a randomized, placebo-controlled, double-blinded and parallel group, single centre study. The clinical study protocol was written in English but was also explained in a language (Hindi and Kannada) the volunteer subjects understood. The Informed consent form (ICF), which also included the study information for the patients, was also printed in Hindi and Kannada and provided to the subjects. Written informed consent was obtained from all subjects. The Committee for Evaluation of Protocols for Clinical Research (CLINICOM), an independent ethics committee in Bangalore, India approved the study (Protocol MA-CT-09-12) on 22 January 2010 in accordance with the ethical principles that have their origins in the Declaration of Helsinki.

**Fig. 1:**
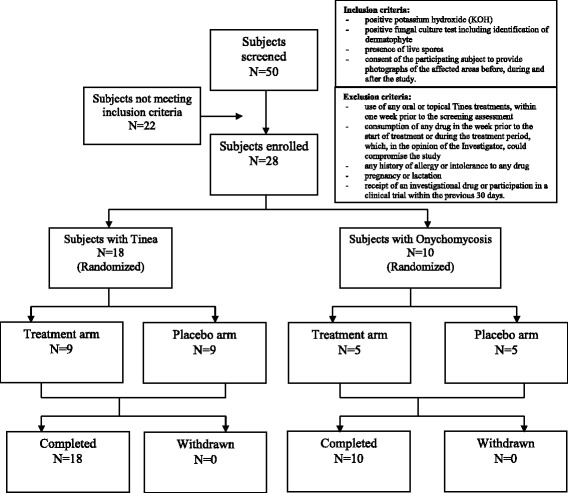
Study disposition

The study was conducted at a single centre at the Apollo Clinic Bangalore, India. Males and females aged ≥18 years, with tinea of combined severity (itching, erythema and scaling) score of ≥8 or with severe or very severe onychomycosis and who had provided the written informed consent and consent for taking photographs of the affected region were enrolled in the study. These subjects were also diagnosed with positive KOH smear, positive fungal culture test with identification of dermatophyte and presence of live spores. Patients who received any oral or topical tinea treatments one week prior to screening or had ingested any drug in the week prior to start or during the treatment were excluded from the study. Other exclusion criteria are summarized in Fig. 1.

The study coordinator (Manipal Acunova) randomized the subjects to either treatment (Calmagen® dermaceutical cream [12% AMYCOT®] or lotion [8% AMYCOT®]) or placebo arm in a 1:1 ratio, according to a computer-generated list by block randomization (Fig. 1). A random number was allocated to each box of study medication and the study site dispensed the study medication in sequential order as the patients qualify for study participation. The study coordinator kept one set of sealed envelopes containing the treatment codes while a second set was kept at the study site with the corresponding study medication. All patients and the investigator involved in conducting the study were blinded to treatment codes. Upon completion of the study, all sealed envelopes were returned to the study coordinator.

Subjects with tinea applied either Calmagen® or placebo cream twice daily to the affected area of skin for a period of four weeks. Subjects with onychomycosis applied either Calmagen® or placebo lotion twice daily over the face of the nail and under and around the margins and cuticle for 12 weeks. An additional observation phase of 12 weeks was undertaken for subjects with onychomycosis, during which the lotion was applied only once daily. After the observational phase (month six), the change in surface area and severity of target lesions in onychomycosis subjects were evaluated.

The primary efficacy endpoint was percentage of subjects who had achieved mycological cure at the end of the study. Mycological cure included assessing KOH smear, fungal culture and live spore counts, by obtaining scrapings from the lesion and analysed with standard methods as previously described [15–17]. Direct microscopic examination of the above specimen was performed to detect fungal spores or hyphae. The investigator assessed the secondary endpoints during scheduled visits. The secondary efficacy endpoints included (i) reduction in size and severity score of target lesion (tinea or onychomycosis), (ii) clinical cure defined as IGA response of ‘cleared’ or ‘excellent’, (iii) improvement in lesions assessed by photographic record. Assessment of Onychomycosis severity scores was based on clinical practice opinions and the Scoring Clinical Index for Onychomycosis (SCIO) [18]. Scoring of the severity of Tinea lesions were based on clinical experience and adapted from Reyes et al. [19]. Safety evaluation included assessment of adverse events and assessment of vital signs.

### Statistical analysis

The sample size for this study was computed based on the results from a previous open label, non-comparative multi-centre study on the 1% oxiconazole cream and lotion anti-fungal [20]. From the aforementioned study (Jerajani et al.), the observed 81% cure rate for the experimental drugs and an assigned 10% IGA response of ‘cleared’ or ‘excellent’ for the placebo group were used as parameters to determine the sample size in the present study using a two-group Fisher’s-exact test of equal proportions. Based on these assumptions, there is over 95% power to detect a significant difference between the Calmagen® and placebo groups with 14 subjects per group (28 subjects overall) at 5% level of significance, and assuming a 20% dropout rate.

All patients who received the study treatment and had at least one efficacy measurement subsequent to the baseline visit, were included in the modified intention-to-treat (mITT) population which was the primary population for efficacy analysis. Patients who completed both the baseline and end of treatment visits and who had no major protocol violations were included in the per-protocol (PP) population, which was the secondary population for efficacy analysis. The primary efficacy endpoint was also analyzed for the per-protocol (PP) population which includes all randomized patients who complete both the baseline visit and end of treatment visit and who have no major protocol violations. Since all subjects completed the study without protocol violations, the PP and mITT populations were essentially the same. All secondary efficacy endpoints were analyzed for the mITT and PP populations. Safety data were analyzed for Safety Population, which included all patients who have received at least one application of study drug.

The parameters for the primary endpoint were analysed using the two proportions (Binomial) test. For the secondary endpoint, analysis of variance (ANOVA) model was used to evaluate the reduction in size and severity score of the target lesion of tinea, or extent and severity of onychomycosis compared to the baseline at the end of the study. Summary of the IGA responses and improvements in lesions assessed by photographic record response of ‘Yes’ or ‘No’ were recorded.

For safety evaluation, all patients who were randomized and dispensed study medication (Intention-to-treat [ITT] population) were included in the analysis. A *p*-value <0.05 was considered significant. The SAS package (SAS® Institute Inc., USA, and Version 9.2) was used for statistical evaluation.

## Results

A total of 50 subjects were screened, of whom 28 (18 with tinea (six patients each of Tinea cruris, corporis and pedis) and ten with onychomycosis) met the inclusion criteria outlined in Fig. 1, and were included in the study. Twenty-two of the screened subjects were excluded since they did not meet the inclusion criteria. They were randomly assigned to either treatment or placebo arms in a ratio of 1:1. The first patient was enrolled on 27 March 2010 and the last patient completed the study on 31 January 2011. There were no withdrawals or dropouts, and all 28 subjects completed the study (Fig. 1). All 28 enrolled subjects were part of the same PP, mITT and ITT populations used for efficacy and safety analysis. The baseline characteristics were similar in both the study arms (Tables 1 and 2).

**Table 1:** Benchmark for secondary efficacy endpoints

A. Tinea lesions scoring^19^		
Score		Description
0		Absent
1		Mild
2		Moderate
3		Severe
4		Very severe
B. Scoring Clinical Index for Onychomycosis (SCIO)^18^		
Score	Grade	Description
0	None	• No infection
1	Mild	• Depth of involvement <1/3 • Scaling/ hyperkeratosis absent or <1 mm • Discolouration of nail
2	Moderate	• Depth of involvement 1/3 to 2/3 • Scaling/ hyperkeratosis 1–2 mm • Acute Paronychia [acute inflammation surrounding nail(s)] • Nail plate dystrophy up to 50%.
3	Severe	• Depth of involvement >2/3 • Scaling/ hyperkeratosis >2 mm • Chronic Paronychia [chronic inflammation surrounding nail(s)] • Nail plate dystrophy >50%
C. Investigator Global Assessment (IGA) response^20^		
Score	Response	Description
5	Cleared	100% remission except residual manifestations
4	Excellent	90–99% improvement
3	Good	50–89% improvement
2	Fair	25–49% improvement
1	Poor	<24% improvement
0	Worse	deterioration from baseline

Tinea lesions scoring [19] (A). Onychomycosis scoring - Scoring Clinical Index for Onychomycosis (SCIO) [18] (B). Investigator Global Assessment (IGA) response [20] (C)

**Table 2:** Baseline characteristics

Parameter	Treatment group (*N* = 14)	Placebo group (*N* = 14)
Age (years; Mean ± SD)	37.2 ± 9.40	45.8 ± 12.26
Gender (Male:Female)	9:5	11:3
Height (cm; Mean ± SD)	165.2 ± 7.06	168.4 ± 5.45
Weight (kg; Mean ± SD)	63.3 ± 8.59	68.0 ± 9.32
Asian Race (N)	14 (100%)	14 (100%)
KOH Smear positive	14 (100%)	14 (100%)
Fungal culture positive	14 (100%)	14 (100%)
Live spore count positive	14 (100%)	14 (100%)
Type of Tinea and causative organism		
Tinea cruris	5	1
Epidermophyton floccosum	3	0
Trichophyton violaceum	1	1
Trichophyton rubrum	1	0
Tinea corporis	2	4
Epidermophyton floccosum	1	1
Trichophyton violaceum	0	1
Trichophyton rubrum	1	2
Tinea pedis	2	4
Epidermophyton floccosum	2	4
Trichophyton violaceum	0	0
Trichophyton rubrum	0	0
Causative organism for Onychomycosis		
Epidermophyton floccosum	1	2
Trichophyton mentagrophytes	2	2
Trichophyton rubrum	2	1
Severity score		
Tinea (Mean ± SD)	9.0 ± 0.5	8.9 ± 0.6
Size of lesion in subjects with Tinea (cm; Mean ± SD)	13.3 ± 5.1	8.0 ± 1.4
Onychomycosis (Mean ± SD)	3.0 ± 0.0	3.0 ± 0.0
Surface area affected in Onychomycosis (%; Mean ± SD)	63.0 ± 4.5	66.0 ± 5.5

*SD* Standard deviation, *cm* Centimetre, *KOH* Potassium hydroxide

### Efficacy

#### Primary endpoint

**Fig. 2:**
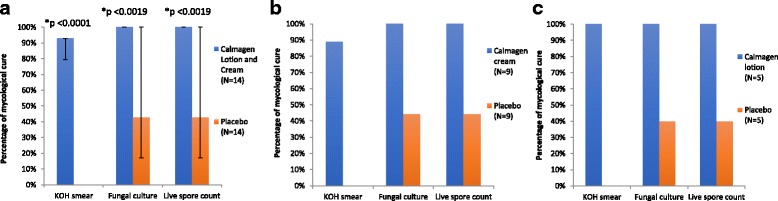
Calmagen® lotion and cream met primary endpoint: mycological cure based on KOH smear, fungal culture and live spore count. **a** Combined results on Calmagen® cream and lotion; **b** Calmagen® cream sub-group results; **c** Calmagen® lotion sub-group results; p-value; *: statistically significant; N: Number of subjects; KOH:Potassium hydroxide; |-|: confidence interval

The primary endpoint of mycological cure from combined tinea and onychomycosis infections was assessed by KOH smear, fungal culture and live spore count (Fig. 2).

At the screening visit, all the 28 (100%) subjects were positive for KOH smear. At the end of the study, 13 of 14 (92.86%) were found negative in the treatment arm while none of the subjects in the placebo arm were cured showing a significant difference between both arms (*p* < 0.0001) (Fig. 2a). Subgroup analysis showed that 8 out of 9 tinea (88.9%) subjects were found negative in the Calmagen® cream treatment arm, but in the placebo arm, 44.4% achieved mycological cure based on KOH smear test (Fig. 2b). On the other hand, 5 out of 5 onychomycosis (100%) subjects treated with Calmagen® lotion and only 40% of the subjects receiving placebo were mycologically cured based on the same parameter (Fig. 2c).

Fungal culture and live spore count were also positive at baseline in all 28 subjects. At the end of the study, it was negative in all 14 subjects in the combined Calmagen® treatment arm, whereas in the placebo arm, only six of 14 (42.9%) had negative culture results (*p* = 0.0019; Fig. 2a). Subgroup analysis showed that 9 out of 9 tinea (88.9%) subjects were found negative in the Calmagen® cream treatment arm, but in the placebo arm, 44.4% achieved mycological cure based on fungal culture and live spore count (Fig. 2b). On the other hand, 5 out of 5 onychomycosis (100%) subjects treated with Calmagen® lotion and only 40% of the subjects receiving placebo were mycologically cured based on the same parameters (Fig. 2c).

At baseline, fungal culture was positive in all tinea and onychomycosis patients of both the treatment and placebo groups (Table 3). *Epidermophyton floccosum* was the predominant organism in the Tinea group (11 of 18, Table 3). *T. mentagrophytes*, *T. rubrum* and *E. floccosum* were identified in the onychomycosis subjects (Table 3). At the end of the study, fungal culture was negative for all the patients treated with Calmagen®.

**Table 3:** Fungal species identified in subjects

Type of infection	Fungal culture at baseline	Fungal culture at end of treatment^a^	Species identified	
			Baseline	End of treatment
Treatment arm (N = 14)				
Tinea cruris	Positive	Negative	*Epidermophyton floccosum*	None
Tinea corporus	Positive	Negative	*Tricophyton rubrum*	None
Tinea pedis	Positive	Negative	*Epidermophyton floccosum*	None
Tinea cruris	Positive	Negative	*Epidermophyton floccosum*	None
Tinea cruris	Positive	Negative	*Tricophyton rubrum*	None
Tinea corporus	Positive	Negative	*Epidermophyton floccosum*	None
Tinea cruris	Positive	Negative	*Tricophyton rubrum*	None
Tinea cruris	Positive	Negative	*Epidermophyton floccosum*	None
Tinea pedis	Positive	Negative	*Epidermophyton floccosum*	None
Onychomycosis	Positive	Negative	*Tricophyton mentagrophytes*	None
Onychomycosis	Positive	Negative	*Tricophyton rubrum*	None
Onychomycosis	Positive	Negative	*Tricophyton rubrum*	None
Onychomycosis	Positive	Negative	*Epidermophyton floccosum*	None
Onychomycosis	Positive	Negative	*Tricophyton mentagrophytes*	None
Placebo group (N = 14)				
Tinea pedis	Positive	Negative	*Epidermophyton floccosum*	None
Tinea pedis	Positive	Positive	*Epidermophyton floccosum*	*Epidermophyton floccosum*
Tinea corporus	Positive	Positive	*Epidermophyton floccosum*	*Epidermophyton floccosum*
Tinea corporus	Positive	Positive	*Tricophyton rubrum*	*Tricophyton rubrum*
Tinea corporus	Positive	Positive	*Tricophyton rubrum*	*Tricophyton rubrum*
Tinea pedis	Positive	Negative	*Epidermophyton floccosum*	None
Tinea pedis	Positive	Negative	*Epidermophyton floccosum*	None
Tinea corporus	Positive	Positive	*Tricophyton rubrum*	*Epidermophyton floccosum*
Tinea cruris	Positive	Negative	*Tricophyton rubrum*	None
Onychomycosis	Positive	Positive	*Tricophyton mentagrophytes*	*Tricophyton mentagrophytes*
Onychomycosis	Positive	Positive	*Epidermophyton floccosum*	*Epidermophyton floccosum*
Onychomycosis	Positive	Positive	*Epidermophyton floccosum*	*Epidermophyton floccosum*
Onychomycosis	Positive	Negative	*Tricophyton rubrum*	None
Onychomycosis	Positive	Negative	*Tricophyton mentagrophytes*	None

^a^Fungal culture at week 4 for tinea and 3 months for onychomycosis

#### Secondary endpoints

**Fig. 3:**
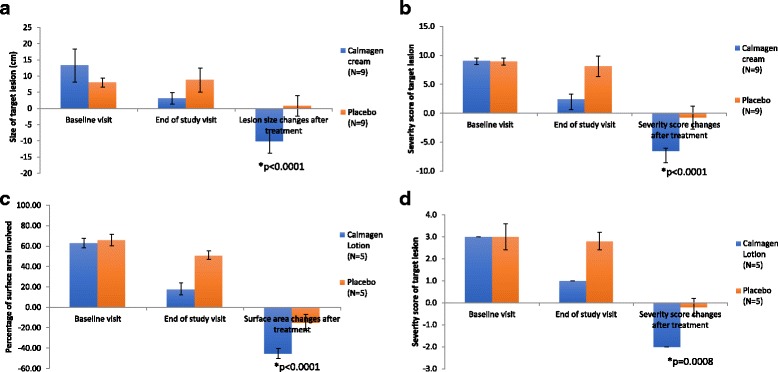
Calmagen® cream and lotion met secondary endpoints: Changes from baseline and treatment in size (**a**) and in target lesion severity score (**b**) for tinea subjects. Changes from baseline and treatment in surface area involved (**c**) and in target lesion severity score (**d**) for onychomycosis subjects. *p*-value; *: statistically significant; N: Number of subjects; cm: centimetre; |-|: standard deviation

In the Calmagen® cream treatment arm, the size of the tinea lesion at baseline and end of the study were significantly reduced in mean size by 10.2 ± 3.7 cm, while there was no significant change observed in the placebo arm (Fig. 3a). There was a significant mean difference of 11.0 cm in the size reduction of the lesion between the treatment and placebo arms at the end of the study (95% CI: −14.47 to −7.53 cm; *p* < 0.0001).

The Calmagen® cream treatment arm had an eight-fold reduction in severity score of tinea lesions from baseline to end of study compared to the placebo arm, which had no significant reduction in tinea lesion severity score observed at the same end points (Fig. 3b). The mean difference in severity score of - 5.78 between the treatment and placebo arms was significant (95% CI:-7.32 to −4.23; *p* < 0.0001).

There was a 45% reduction in percentages of affected surface area for onychomycosis after Calmagen® lotion treatment, almost three-fold higher than observed in the placebo arm (−15.0%), a − 30.0% mean difference, which is significant (Fig. 3c; 95% CI: −39.65% to −20.35%; *p* < 0.0001). In terms of severity scores of onychomycosis (Fig. 3d), a very significant 10-fold difference in degree of reduction or mean difference of −1.8 in severity score, was observed in the treatment arm compared to the placebo arm (2.0 ± 1.0 vs 0.2 ± 0.4, respectively; 95% CI: −2.36 to −1.24; *p* = 0.0008).

**Fig. 4:**
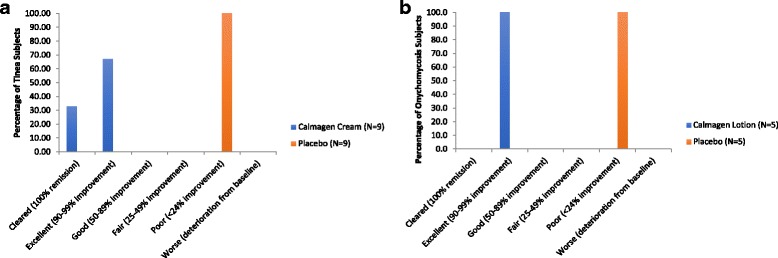
Calmagen® cream and lotion met secondary endpoint: IGA Response. Summary IGA response of tinea (**a**) and onychomycosis (**b**) subjects. N: Number of subjects

At the end of the study, in the treatment arm of tinea subjects, IGA response of ‘cleared’ was achieved in three (33.33%) and ‘excellent’ in six (66.67%) of nine subjects (Fig. 4a), while in the onychomycosis treated subjects, a response of ‘excellent’ was achieved in all five subjects (100%) (Fig. 4b). In the placebo arms of both tinea and onychomycosis subjects, IGA response of ‘cleared’ or ‘excellent’ was not achieved in any of the subjects and all subjects did not show any improvement from baseline (Fig. 4a-b).

Photographic record at the end of the study visit showed improvement in all subjects with tinea lesions as well as onychomycosis in the treatment arm, while in the placebo arm, none of the subjects showed any improvement (data not shown).

#### Safety

There were no adverse events reported in the treatment arm, while in the placebo arm, one patient reported pain in both the legs, which was mild and not related to the study medication.

## Discussion

The aim of the current study was to establish the fungicidal profile, safety and efficacy of Calmagen® dermaceutical cream and lotion as topical treatment in patients with severe to very severe presentations of tinea and onychomycosis.

*Epidermophyton floccosum* was the predominant organism identified in the tinea group, which is known to be associated with the infection. Previous studies have reported *T. rubrum* [23, 24] and *T. interdigitale* [21] to be the most commonly isolated causative organism in onychomycosis while *T. mentagrophytes*, *T. rubrum* and *E. floccosum* were identified in the onychomycosis subjects of this study.

Mycological and clinical cures were achieved in most subjects treated with Calmagen® with no adverse side effects. This study confirms findings of a previous investigator-initiated open label study, which showed that Calmagen® lotion (8% AMYCOT®) is effective in the management of onychomycosis [14]. A total of 10 patients with onychomycosis were treated with Calmagen® lotion topically twice daily for three months. At the end of three months, 7 out of 10 subjects had 100% clearance of the lesion. Of the remaining three, one showed 100% clearance at the six-month follow-up visit, while the other two subjects declined follow-up.

There was a male preponderance in our study (71.4%). In a study on subjects with onychomycosis, a similar male:female ratio of 6.7:1was observed [22]. However, in other studies, females seemed to be more commonly affected (61.4%) [2, 23]. The mean age of our study’s population was 41.5 years. In one study, the most prevalent age group was 51–60 years [2] and 42.4 years in another study [21].

Efficacy has been reported previously for other antifungal treatments of mild cases but without the safety profile exhibited by Calmagen®. Calmagen® cream and lotion formulations are based on cosmetic excipients registered with INCI (International Nomenclature of Cosmetic Ingredients), which makes application of Calmagen® cream and lotion easier on the skin and nail without any adverse reactions. In contrast, most of the chemical-based anti-fungals have reported skin irritations as a commonly reported adverse event. In patients with *Tinea cruris*, complete clearance was observed in 21.2% treated with luliconazole cream 1% [25]. In another study, Global Evaluation Response revealed that skin lesions were completely cleared with Whitfield’s ointment + oral fluconazole and butenafine cream in 98% patients with tinea. However, Whitfield’s ointment caused burning and redness, oral fluconazole caused gastritis, while no adverse events were reported with topical butenafine [26]. Terbinafine and sertaconazole resulted in complete cure in 100% subjects with *Tinea cruris* and *Tinea corporis* at the end of 3 weeks of treatment [27]. While both topical as well as oral formulations of antifungal drugs are available, topical treatments are preferred in superficial tinea because of high cure rates with 2–4 weeks of therapy. Additionally, systemic absorption with topical drugs is minimal, and adverse effects are limited to skin reactions at the site of application, which are usually mild and transient. However, oral antifungal drugs such as terbinafine can cause serious adverse effects like hepatotoxicity and Stevens–Johnson syndrome [8].

Onychomycosis is more challenging to treat compared to other superficial dermatophytoses, because of difficulties in drug penetration through the nail. The US FDA (United States Food and Drug Administration) approved tavaborole and efinaconazole for topical treatments against mild-to-moderate onychomycosis in 2014. In two phase III studies on 10% efinaconazole topical solution, mycological cure rates of 55.2% and 53.4% and complete cure rates of 15.2% and 17.8% at week 52, were observed [28]. In another efinaconazole study, a mycological cure rate of 56% and clinical treatment success of 43% were observed after week 52 [29]. According to Canadian guidelines, efinaconazole is recommended only in treating mild and moderate onychomycosis, but not in severe cases (where terbinafine is preferred) [30]. In two phase III studies on 5% tavaborole topical solution, mycological cure was observed in 31.1–35.9% and complete cure of only 7–9% at week 52. Application-site reactions were the most common side-effects and included exfoliation (2.7%), erythema (1.6%), and dermatitis (1.3%) [31].

Our study has some limitations. The study had a small sample size and did not permit stratification by treatment (lotion or cream), organism type, type of fungal infection and infection severity. Furthermore, there was no washout period or a period of complete cessation of treatment to ensure that the fungal infections did not recur. However, the present study forms the foundation for a larger clinical trial that will address these limitations.

### Post study evidence

**Fig. 5:**
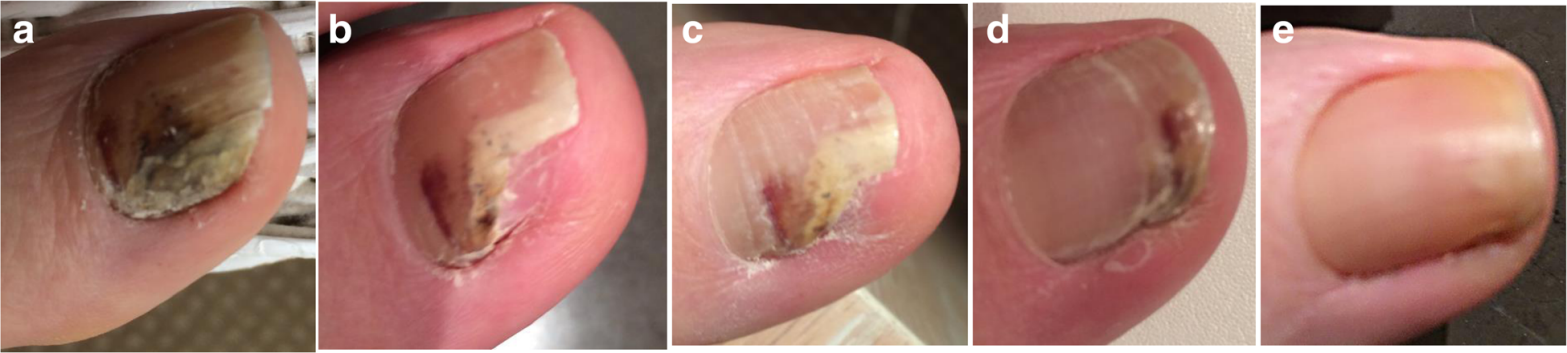
Case 1: Calmagen® lotion cleared onychomycosis in a 50+ year-old Caucasian male who had the condition for 4 years and tried different treatments which failed. Before treatment (**a**); 1 month after treatment (**b**); 2 months after treatment (**c**); 8 months after treatment (**d**); 13 months after treatment and 2 months after stopping treatment (**e**)

**Fig. 6:**
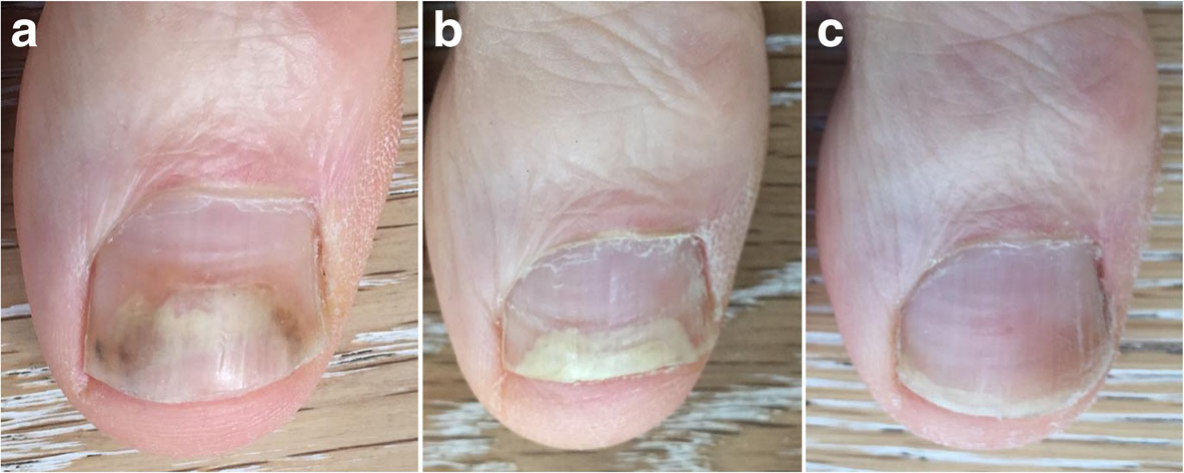
Case 2: Calmagen® lotion cleared onychomycosis in a 50+ year-old Caucasian male who had the condition for 10 years and tried different treatments including oral terbinafine which failed. Before treatment (**a**); 1.5 months after treatment (**b**); 14 months after treatment and 11 months after stopping treatment (**c**)

After completion of this study, there have been numerous testimonials and anecdotal evidence on the efficacy of the Calmagen® lotion and cream. This is evident in two cases of onychomycosis in two Caucasian males who had the problem for several years. Both individuals had unsuccessfully tried several anti-fungal treatments, but their conditions were only successfully treated with Calmagen® lotion with no recurrence after at least 2 months from clearance of the infections (Figs. 5 and 6).

## Conclusions

Most anti-fungal agents are chemical based and like the emerging laser-based treatment, have significant side effects. Although Calmagen® has shown promising efficacy and safety, there is a need for a larger multi-centre, randomized trial for each formulation of Calmagen®, benchmarking it against other standard antifungal drugs. Nevertheless, this study confirms that Calmagen® cream and lotion containing AMYCOT® are potentially safe and efficacious, representing a natural alternative for the management of severe to very severe tinea skin and fungal nail (onychomycosis) infections.

## Abbreviations

ANOVA: Analysis of variance
CI: Confidence interval
Cm: Centimetre
IGA: Investigator Global Assessment
INCI: International Nomenclature of Cosmetic Ingredients
ITT: Intention-to-treat
KOH: Potassium hydroxide
MIC: Minimum inhibitory concentration
mITT: Modified intention-to-treat
SCIO: Scoring Clinical Index for Onychomycosis
SD: Standard deviation
USFDA: United States Food and Drug Administration
